# Inhibition of Hepatitis B Virus and Induction of Hepatoma Cell Apoptosis by ASGPR-Directed Delivery of shRNAs

**DOI:** 10.1371/journal.pone.0046096

**Published:** 2012-10-19

**Authors:** Jingwei Ma, Chunmei Huang, Xinxin Yao, Chuan Shi, Lifang Sun, Lu Yuan, Ping Lei, Huifen Zhu, Hongbo Liu, Xiongwen Wu, Qin Ning, Chun Zhou, Guanxin Shen

**Affiliations:** 1 Department of Immunology, Tongji Medical College, Huazhong University of Science and Technology, Wuhan, People's Republic of China; 2 Department of Infectious Disease, Tongji Hospital, Tongji Medical College, Huazhong University of Science and Technology, Wuhan, People's Republic of China; 3 Department of Environmental Health Sciences, Columbia University, New York, New York, United States of America; University Hospital of Essen, Germany

## Abstract

Hepatitis B virus (HBV) infection is a worldwide liver disease and nearly 25% of chronic HBV infections terminate in hepatocellular carcinoma (HCC). Currently, there is no effective therapy to inhibit HBV replication and to eliminate hepatoma cells, making it highly desired to develop novel therapies for these two stages of the HBV-caused detrimental disease. Recently, short hairpin RNA (shRNA) has emerged as a potential therapy for virus-infected disease and cancer. Here, we have generated a shRNA, pGenesil-siHBV4, which effectively inhibits HBV replication in the human hepatoma cell line HepG2.2.15. The inhibitory effects of pGenesil-siHBV4 are manifested by the decrease of both the HBV mRNA level and the protein levels of the secreted HBV surface antigen (HBsAg) and HBV e antigen (HBeAg), and by the reduction of secreted HBV DNA. Using mouse hydrodynamic tail vein injection, we demonstrate that pGenesil-siHBV4 is effective in inhibiting HBV replication *in vivo*. Because survivin plays a key role in cancer cell escape from apoptosis, we further generated pGenesil-siSurvivin, a survivin-silencing shRNA, and showed its effect of triggering apoptosis of HBV-containing hepatoma cells. To develop targeted shRNA therapy, we have identified that as a specific binder of the asialoglycoprotein receptor (ASGPR), jetPEI-Hepatocyte delivers pGenesil-siHBV4 and pGenesil-siSurvivin specifically to hepatocytes, not other types of cells. Finally, co-transfection of pGenesil-siHBV4 and pGenesil-siSurvivin exerts synergistic effects in inducing hepatoma cell apoptosis, a novel approach to eliminate hepatoma by downregulating survivin via multiple mechanisms. The application of these novel shRNAs with the jetPEI-Hepatocyte targeting strategy demonstrates the proof-of-principle for a promising approach to inhibit HBV replication and eliminate hepatoma cells with high specificity.

## Introduction

Hepatitis B virus (HBV) is a partially double-stranded DNA virus. Affecting 400 million people worldwide, chronic HBV infection is a devastating liver disease. Moreover, chronic HBV carriers have approximately 100 times more chance of having hepatocellular carcinoma (HCC) than normal individuals, known as HBV-related hepatocarcinogenesis [1]. Indeed, nearly 25 percent of chronic HBV carriers can terminate in HCC [2], [3], [4]. Available medicine for chronic HBV infection includes cytokines, such as interferon-alpha, and nucleoside/nucleotide analogues, such as lamivudine and adefovir. However, these treatments cause serious side effects and selection of resistant virus mutations, yielding ineffective therapy. Furthermore, there is currently no cure for HCC. Thus, novel therapeutic approaches are needed to counteract HBV infection and HCC.

RNA interference (RNAi) has been established as an effective way to downregulate gene expression by degrading specific mRNAs [5]. Unlike synthetic small interfering RNAs (siRNA), short hairpin RNA (shRNA) is to use mammalian expression vector to express siRNA inside cells, rendering higher efficacy of targeted mRNA degradation [6]. For example, we have generated effective shRNAs to inhibit melanoma growth [7]. As shRNAs have also been shown to be able to inhibit HBV replication [8], [9], [10], we set out to test the principle of utilizing shRNA approach to address two key issues in chronic HBV infection and its related HCC: HBV replication and unlimited tumor cell growth.

Survivin belongs to the protein family of inhibitor of apoptosis protein (IAP) and has been found to suppress apoptosis and play a key role in cell division [11]. Notably, survivin is abundantly expressed in most human tumor cells, including hepatocellular cancer cells, but barely detectable in normal differentiated cells [11], [12]. This differential expression of survivin in cancer versus normal tissue makes it a promising therapeutic target. In fact, downregulation of survivin expression by siRNAs or hammerhead ribozymes has resulted in increase of tumor cell apoptosis [13], [14]. Although inhibition of survivin by shRNA may induce hepatoma cell apoptosis, to date, there are very limited reports of experimental evidence to support this notion [15]. On the other hand, the HBV protein HBx has been reported to form a protein complex with the hepatitis B virus X-interacting protein (HBXIP) and survivin [16] and to increase the protein level of survivin [17], [18], [19]. Consequently, HBx inhibits hepatoma cell apoptosis via survivin [16], [17], [19]. Thus, through HBx, survivin is involved in the mechanism for HBV to induce hepatoma development.

Targeted delivery is critical for applying shRNA as therapeutics in clinical treatment. However, specific delivery method to deliver shRNA into liver cells has not been extensively explored. It has been shown that cell-receptor mediated endocytosis is an efficient way to deliver shRNA into the targeted cells with high specificity [7], [20]. Hepatocytes express the unique asialoglycoprotein receptors (ASGPR), known as hepatic lectins, which clear desalinated galactose-terminal glycoproteins from the circulation by receptor-mediated endocytosis [21]. Using polymeric carriers with ligands such as galactose, several research groups have promoted specific cellular uptake via receptor-mediated endocytosis in human liver cells [20], [22].

In the present study, we have generated two effective shRNAs that inhibit HBV replication and induce apoptosis of hepatoma cells, and established specific shRNA delivery for hepatic cells using jetPEI-Hepatocyte; the latter is a galactose-conjugated linear polyethylenimine derivative that facilitates transfection to cells that bear ASGPR receptors. The data show that HBV replication is inhibited by the shRNA pGenesil-siHBV4 both *in vitro* in HepG2.2.15 cells and *in vivo* in an acute HBV infection mouse model. We also show that another shRNA, pGenesil-siSurvivin, induces apoptosis of HBV-positive hepatoma cells. In addition, we demonstrate that jetPEI-Hepatocyte mediates specific shRNA transfection to hepatocytes, not other types of cells, thereby providing a targeted shRNA delivery. Importantly, we identified a new approach to maximize the induction of hepatoma cell apoptosis through the synergistic effects of pGenesil-siSurvivin and pGenesil-siHBV4. Those results establish a proof-of-principle for a promising shRNA approach to treat chronic HBV infection and its transformed hepatocellular carcinoma.

## Results

### Generation of effective HBV shRNA

**Figure 1:**
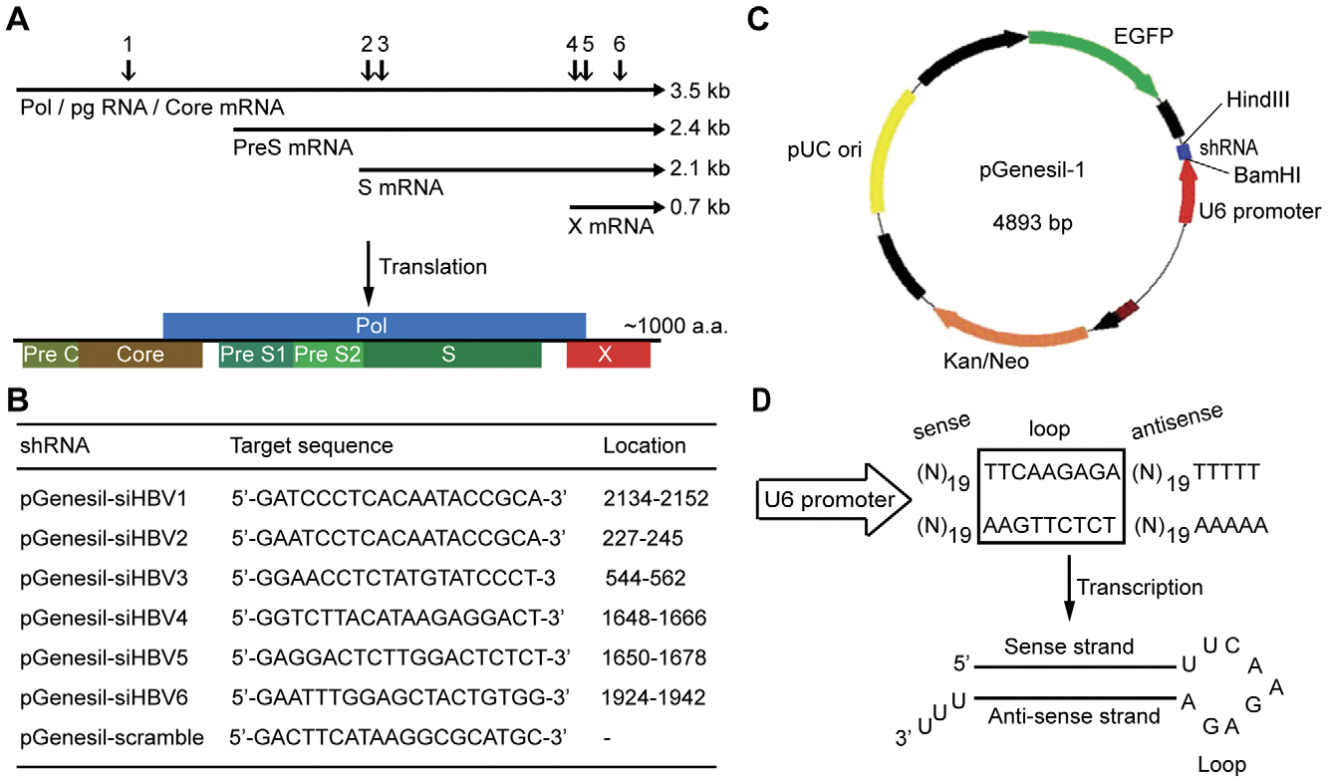
Construction of HBV shRNAs. (**A**) Six target sequences for generating shRNAs are selected from the HBV genome. Arrows indicate the locations of the target sites within the four HBV transcripts. The corresponding HBV proteins that are translated from the HBV mRNAs are indicated below. (**B**) The target sequences of the 6 shRNAs (pGenesil-siHBV1∼6) and their locations in the HBV genome, together with the scramble shRNA sequence. (**C**) Schematic of the pGenesil-1 vector. EGFP and inserted shRNA oligonucleotide are transcribed separately. (**D**) Production of shRNA. The DNA oligonucleotide is composed of two 19-nucleotide target sequences in sense and anti-sense separated by a short spacer and is inserted between the BamHI and HindIII restriction sites of pGenesil-1. After transcription driven by the U6 promoter, the shRNA strand is predicted to fold back to form a hairpin RNA, which degrades its targeted mRNA.

The genome of HBV (GenBank accession number: U95551) contains four overlapping open reading frames (ORFs), which encode the viral core protein, e antigen, surface antigen, reverse transcriptase (RT) and HBx protein (Figure 1A). To increase the likelihood of generating effective HBV shRNA, we made 6 shRNA candidates that target various HBV genes required for HBV protein expression and viral replication, including the Core, polymerase-reverse transcriptase (Pol), S and X genes (Figure 1A and 1B). We then subcloned these DNA oligonucleotides into the mammalian expression vector pGenesil-1 (Figure 1C), respectively. pGenesil-1 harbors the U6 promoter to produce shRNA and expresses EGFP as a marker protein to indicate shRNA production inside cells. Based on our preliminary experiments, we designed the gene-specific insert for shRNA that consists of a 19-nucleotide sequence in sense derived from the target gene region, a short spacer (TTCAAGAGA), and the reverse complement antisense sequence of the 19-nucleotides (Figure 1D).

**Figure 2:**
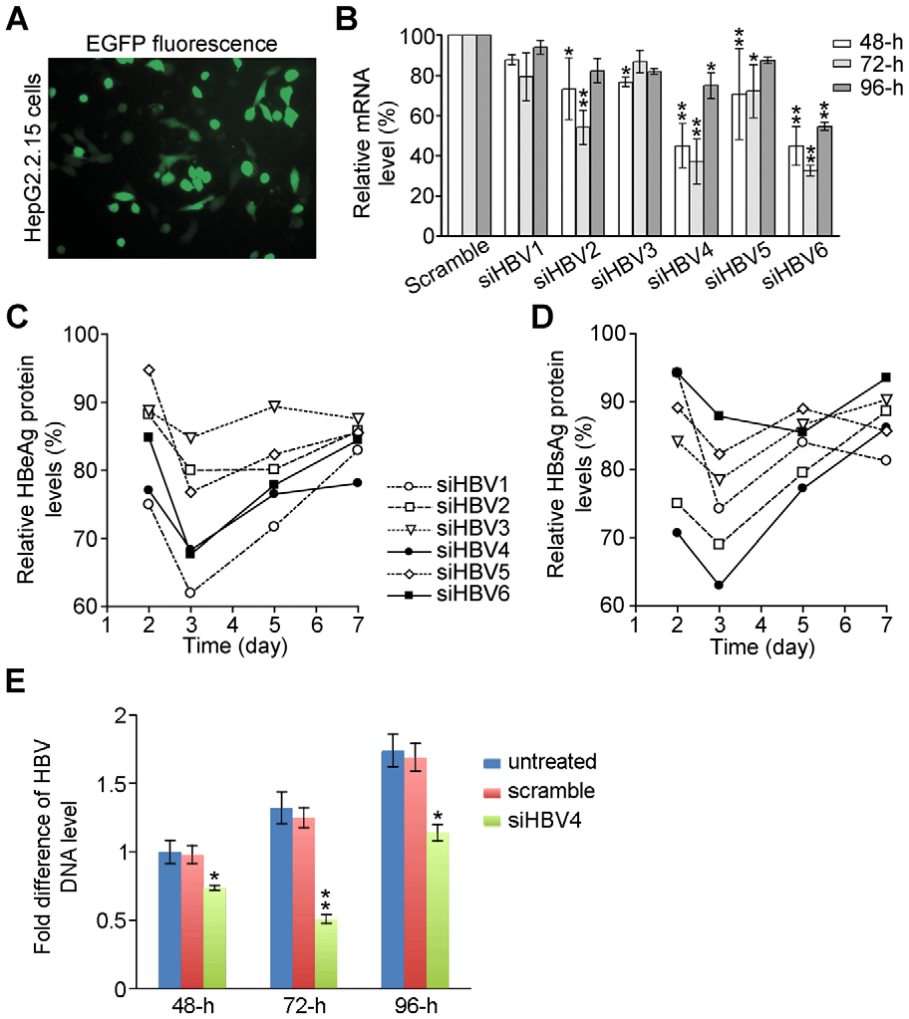
*In vitro* inhibitory effects of the HBV shRNAs on HBV replication in HepG2.2.15 cells. HepG2.2.15 cells were transfected with pGenesil-siHBV1∼6 respectively. (**A**) A representative image of EGFP-positive cells at 24-h post-transfection. (**B**) Silencing effects of pGenesil-siHBV1∼6 on HBV mRNA. Evaluated by real-time PCR at day 2, 3 and 4 post-transfection, the HBV mRNA levels relative to those in scramble shRNA-transfected cells are shown as mean ± SD of three independent experiments. Significant differences compared to the mRNA levels in scramble shRNA-transfected cells are indicated by * (P<0.01) and ** (P<0.001). (**C and D**) The levels of the HBeAg and HBsAg proteins in the culture medium at day 2, 3, 5 and 7 post-transfection. The HBeAg and HBsAg levels were detected by ELISA and normalized to the levels of the cells transfected with the scramble shRNA. Data are shown as mean values of three independent experiments. (**E**) The levels of HBV DNA in the culture medium at 48-h, 72-h and 96-h post-transfection. The cells were transfected with the scramble and siHBV4 shRNAs, respectively. The measured HBV DNA levels were normalized to the level of the untreated cells at 48-h, shown as mean ± SD of three independent experiments. Compared to no treatment and transfection with the scramble shRNA, the siHBV4 transfection induced significant decrease of HBV DNA at all three time points with most effects at 72-h post-transfection (*: P<0.01; **: P<0.001).

To test if these shRNAs are effective in inhibiting HBV replication, we used HepG2.2.15 cells as a cellular model of HBV infection and its related HCC. HepG2.2.15 cells are a human hepatoma cell line that has several copies of the HBV genome inserted into its own genome. Thus, HepG2.2.15 cells stably produce HBV mRNAs, antigens and viral particles [23]. We transfected HepG2.2.15 cells with 6 shRNA plasmids, respectively, using the transfection reagent Lipofectamine 2000, and detected EGFP expression at 24 hours post-transfection (Figure 2A). The transfection efficacy in HepG2.2.15 cells is 31.9%±1.43% (mean ± SD). This transfection efficiency seems specific to HepG2.2.15 cells, as we routinely get higher efficiency in other common cell lines, such as HEK 293 cells (Figure S1). The expression of EGFP suggests production of these shRNAs in HepG2.2.15 cells. So, we tested whether these shRNAs, once produced inside HepG2.2.15 cells, could affect HBV mRNA levels. We isolated the total RNA on day 2, 3 and 4 post-transfection and used real-time PCR to quantify the levels of the corresponding targeted HBV mRNAs (Table S1). When compared to the scramble shRNA, these HBV shRNAs show inhibitory effects on the HBV mRNA levels (Figure 2B). Among them, the HBV shRNAs #4 and #6 (pGenesil-siHBV4 and pGenesil–siHBV6) are more effective than others. In addition, these shRNAs reached to their maximum inhibitory effects at day 3 post-transfection.

We then tested the effects of these shRNAs on viral antigen secretion, an indicator of HBV replication. Using enzyme-linked immunosorbent assay (ELISA), we measured the HBe and HBs protein concentrations in the culture media of shRNA-transfected HepG2.2.15 cells at day 2, 3, 5 and 7 post-transfection, respectively. At day 3 post-transfection, the HBV shRNAs reduced the secreted HBe and HBs protein levels significantly (Figure 2C and 2D). This reduction at the protein levels is consistent with the time course of the inhibitory effects on the mRNA levels caused by the HBV shRNAs. Among these shRNAs, pGenesil-siHBV1, -siHBV4 and -siHBV6 are more effective in decreasing the HBeAg protein level. On the other hand, pGenesil-siHBV2 and -siHBV4 are effective in inhibiting the HBsAg level. Interestingly, for an unknown reason, pGenesil-siHBV1 caused reduction of the HBeAg protein, but not the HBV core mRNA. Nonetheless, these data showed that pGenesil-siHBV4 has higher efficacy in inhibiting HBV amplification at both mRNA and protein levels. Thus, we used pGenesil-siHBV4 for further studies.

To confirm that the HBV virion production is inhibited by pGenesil-siHBV4, we detected the HBV DNA levels in the cell culture medium. As shown in Figure 2E, the HBV DNA in the medium of untreated HepG2.2.15 cells was detectable and its amount increased in a time-dependent manner, indicating the release of the HBV virions from HepG2.2.15 cells. Treatment with the scramble shRNA, pGenesil-scramble, did not affect the levels of HBV DNA at 48-h, 72-h and 96-h post-transfection. However, application of pGenesil-siHBV4 resulted in significant decrease of the HBV DNA level at all the time points measured. Together, the data indicate that the shRNA pGenesil-siHBV4 downregulates the HBV mRNA level, thereby inhibiting HBV virion production.

### *In vivo* inhibition of HBV replication in an acute mouse HBV infection model

**Figure 3:**
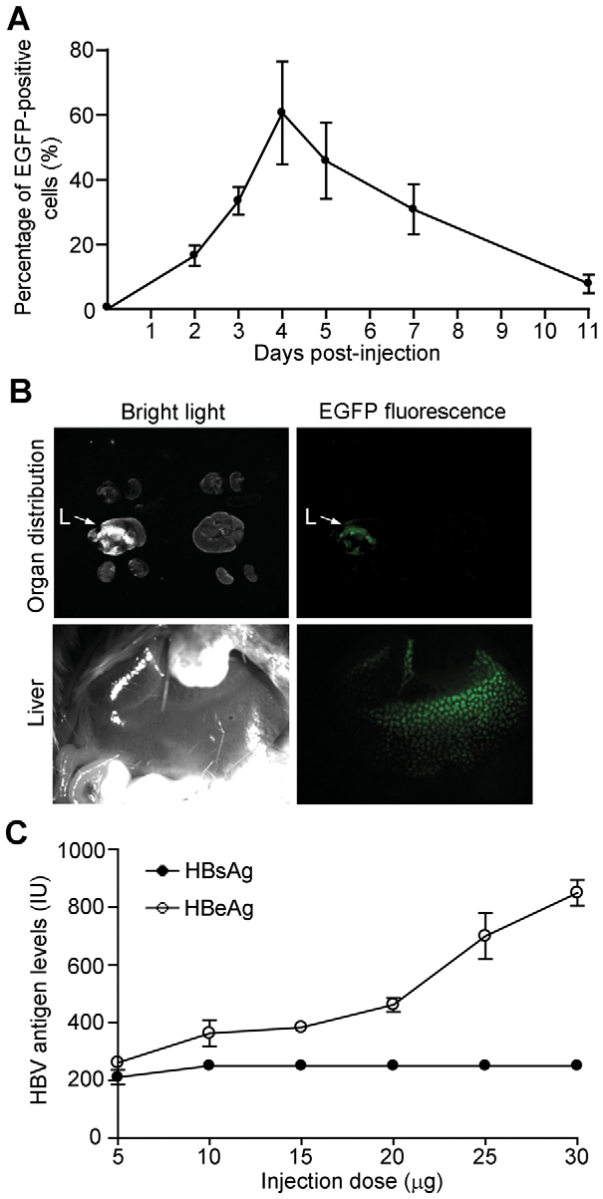
Generation of an acute mouse HBV infection model. (**A**) Time course of EGFP expression in hepatocytes isolated from mice given pGenesil-1 through hydrodynamic tail vein injection. EGFP-positive cells were detected by flow cytometry at day 0, 2, 3, 5, 7 and 11 post-injection. (**B**) EGFP expression in the internal organs of mice given pGenesil-1 as in (A). Images of mouse internal organs were taken at day 4 post-injection by stereoscopic microscopy. Imaged under white light, the left panels show the positions of internal organs: lung, spleen, liver (L) and kidney in the upper left panel; and liver in the lower left panel. The right panels are the same fields as their corresponding left panels, but imaged with 488 nm excitation, to show EGFP fluorescence signals in the organs. (**C**) HBsAg and HBeAg protein levels in the serum of mice given various doses of pBluescript-HBV through hydrodynamic tail vein injection. The protein levels were detected by ELISA at day 4 post-injection. Data in (A) and (C) are shown as mean ± SD of three independent experiments.

To determine whether pGenesil-HBV4 could function *in vivo*, we applied hydrodynamic tail vein injection for both establishing an acute mouse HBV infection model and delivering shRNAs to the liver. After hydrodynamic tail vein injection with the EGFP-expressing pGenesil-1 plasmid, we measured EGFP-positive cells in mouse liver using flow cytometry. The hepatocytes from mice injected with pGenesil-1 have EGFP signal from day 2 to day 11 post-injection, with maximum EGFP expression from day 3 to day 7 (Figure 3A). Crucially, the EGFP signals in the liver are far stronger than other internal organs (Figure 3B), confirming that the hydrodynamic tail vein injection is a useful tool to deliver plasmid DNA specifically to the liver. We then performed hydrodynamic tail vein injection of pBluescript-HBV, a plasmid containing the full length HBV genomic DNA. In the dose-response experiment, after injection of pBluescript-HBV, the HBeAg level in serum increased gradually along the quantity increase of the injected HBV plasmid (Figure 3C). The HBsAg level also increased to 211 IU in the mouse serum of the 5 µg group, but stayed at 250 IU from the 10 µg to 30 µg groups (Figure 3C). Of note, the maximum detection of ARCHITECT i2000SR is 250 IU. Even when we diluted the serum before analysis, HBsAg still remained over the maximum detection level (250 IU), indicating that the level of HBsAg in the serum had reached much higher than 250 IU. Thus, the levels of HBV proteins in the serum of this acute HBV infection mouse model are even higher than those in HBV patient's serum. Together, the data suggest *in vivo* HBV replication in this acute mouse HBV infection model.

**Figure 4:**
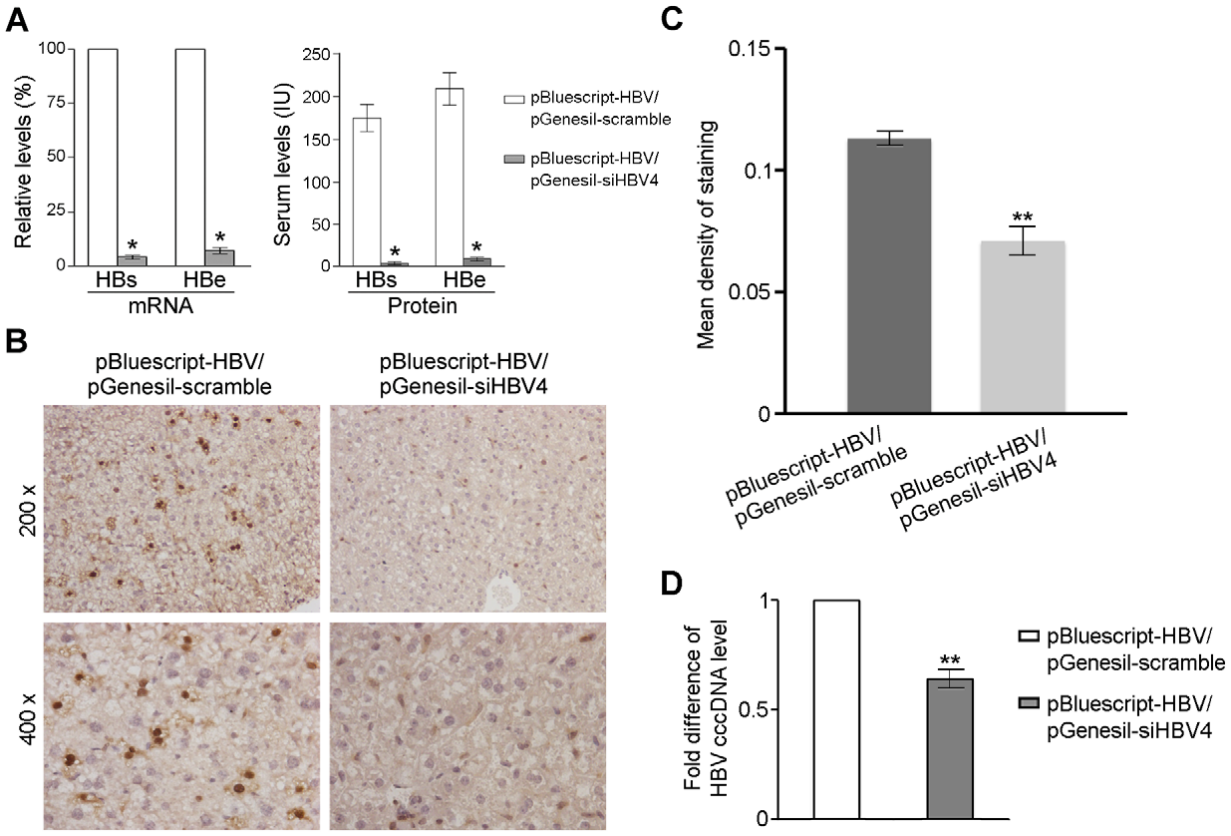
*In vivo* inhibition of HBV replication by pGenesil-siHBV4. pBluescript-HBV/pGenesil-scramble and pBluescript-HBV/pGenesil-siHBV4 plasmids were injected to mice through hydrodynamic tail vein, respectively. (**A**) The mRNA levels of HBsAg and HBeAg in hepatocytes and the protein levels in serum were assayed at day 4 post-injection. The mRNA levels in pBluescript-HBV/pGenesil-siHBV4-treated mice are normalized to their levels in mice treated with pBluescript-HBV/pGenesil-scramble. The protein levels were detected by TRFIA (unit: IU). Data are shown as mean ± SD of three independent experiments. Significant differences compared to the levels in mice injected with pBluescript-HBV/pGenesil-scramble are indicated by * (P<0.01). (**B**) Immunohistochemistry of the mouse liver tissues using the anti-HBcAg antibody at day 4 post-injection. (Original magnification ×200 for the images in the upper panels and original magnification ×400 for the images in the lower panels.) (**C**) Mean density of staining of the HBc protein in the mouse liver tissues described in (B). Random fields were used to calculate the mean densities by the Image-Pro Plus software. Data are shown as mean ± SD of three independent experiments (**: P<0.001). (**D**) HBV cccDNA levels in the liver tissue. Data are normalized to the HBV cccDNA level in the mice injected with pBluescript-HBV/pGenesil-scramble and shown as mean ± SD of three independent experiments (**: P<0.001).

Next, we used this model to assess the *in vivo* efficacy of pGenesil-siHBV4. We performed hydrodynamic tail vein injection with pBluescript-HBV/pGenesil-scramble and pBluescript-HBV/pGenesil-siHBV4 plasmids, respectively. At day 4 post-injection, we quantified the mRNA levels of HBsAg and HBeAg in the liver tissue with real-time PCR. The data were analyzed with comparative delta-delta Ct. Compared to the pBluescript-HBV/pGenesil-scramble group, the pBluescript-HBV/pGenesil-siHBV4 group has significant decrease of both the HBsAg and HBeAg mRNA levels (Figure 4A). Thus, pGenesil-siHBV4 is functional *in vivo* in inhibiting expression of the HBV genes at the mRNA level.

To test if the above *in vivo* inhibition of HBV at the mRNA level could lead to decreased levels of viral proteins, we quantified the serum HBsAg and HBeAg proteins using time-resolved fluoroimmunoassay (TRFIA). In the pBluescript-HBV/pGenesil-siHBV4 group, the serum levels of both proteins reduced, when compared to their levels in the pBluescript-HBV/pGenesil-scramble group (Figure 4A). Thus, not only pGenesil-siHBV4 inhibits HBV at the mRNA level, but also it results in decrease of the HBV protein level. To verify the *in situ* inhibitory effects, we performed immunohistochemistry on the mouse liver tissue to detect HBcAg expression. As expected, in the pBluescript-HBV/pGenesil-scramble group, we observed many HBcAg-positive cells (Figure 4B). At higher magnification (400×), we confirmed that the HBcAg localizes to the cytoplasm of hepatocytes. In the pBluescript-HBV/pGenesil-siHBV4 group, however, HBcAg-positive cells are scarce. With quantification, we show that the hepatocytes in the pBluescript-HBV/pGenesil-siHBV4 group have significant less staining of HBcAg than the cells in the pBluescript-HBV/pGenesil-scramble group (Figure 4C).

Because the HBV covalently closed circular DNA (cccDNA) is a critical HBV replicative intermediate in hepatocytes and plays a key role in viral persistence and reactivation, we analyzed the cccDNA levels in mouse liver tissue. As expected, we detected the HBV cccDNA in the liver tissue of mice injected with pBluescript-HBV/pGenesil-scramble, confirming the HBV replication in this HBV infection model. On the other hand, in the mice injected with pBluescript-HBV/pGenesil-siHBV4, we detected significantly less HBV cccDNA (Figure 4D). Thus, via various measurements, we conclude that the shRNA pGenesil-siHBV4 effectively inhibits HBV replication *in vivo* in the liver.

### Generation of shRNA to induce hepatoma cell apoptosis

For HBV-related HCC, effective therapy needs to be established to remove HBV-transformed hepatoma cells. Toward this goal, we thought to generate shRNA that could induce apoptosis of HBV-transformed tumor cells. We chose to target survivin, an anti-apoptosis factor abundant in most human tumors but not in normal cells, and generated an anti-survivin shRNA pGenesil-siSurvivin.

**Figure 5:**
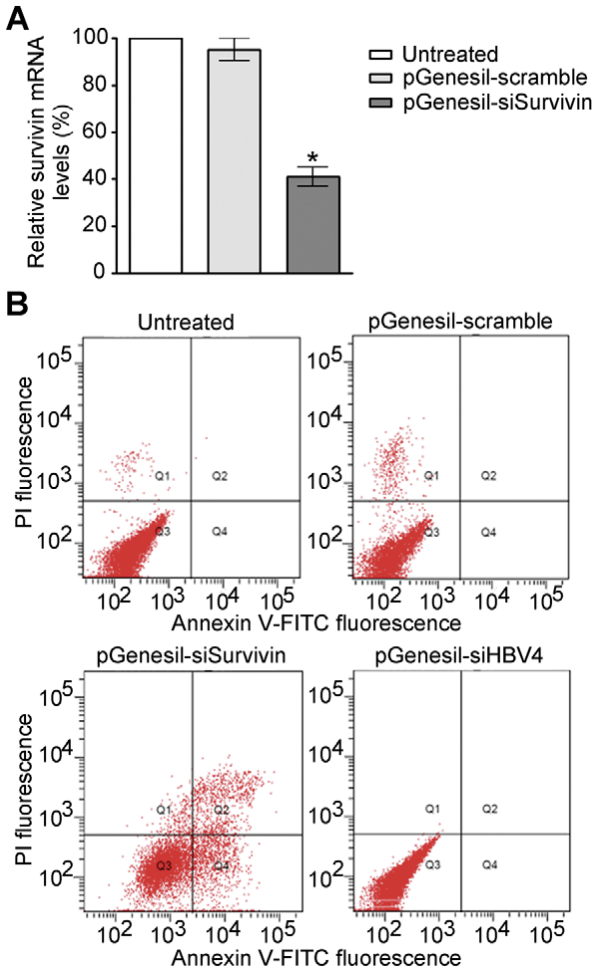
pGenesil-siSurvivin decreases the survivin mRNA level and induces apoptosis of HepG2.2.15 cells. HepG2.2.15 cells were transfected with pGenesil-scramble, pGenesil-siSurvivin and pGenesil-siHBV4, respectively. (**A**) The survivin mRNA levels in transfected cells at day 2 post-transfection. The mRNA levels are normalized to the level in untreated cells. Data are shown as mean ± SD of three independent experiments. Significant difference compared to the mRNA level in untreated cells is indicated by * (P<0.01). (**B**) Annexin V-FITC and PI positive cells detected by flow cytometry at day 2 post-transfection. In the cells transfected with pGenesil-siSurvivin, 17.9% of the cells undergo early-stage of apoptosis (in region Q4) and 14.4% are in late-stage of apoptosis (in region Q2).

To test the efficacy of pGenesil-siSurvivin in reducing the mRNA level of survivin, we transfected HepG2.2.15 cells with pGenesil-scramble and pGenesil-siSurvivin, respectively. As shown in Figure 5A, at 48 hours post-transfection, the survivin mRNA level decreased significantly to 40.98% in pGenesil-siSurvivin transfected cells, compared to the level in the cells without shRNA treatment. The specificity of pGenesil-siSurvivin is demonstrated by the fact that pGenesil-scramble is unable to reduce the survivin mRNA level (Figure 5A). We then detected the effect of pGenesil-siSurvivin in inducing apoptosis of HepG2.2.15 cells, using annexin V-FITC/propidium iodide (PI) double staining. The flow cytometry data show that 32.3% of cells underwent apoptosis at 48 hours after pGenesil-siSurvivin transfection (Figure 5B). On the contrary, untransfected cells and the cells transfected with pGenesil-scramble had no significant apoptosis. Moreover, transfection with pGenesil-siHBV4 is also not able to induce apoptosis of HepG2.2.15 cells, further confirming the specificity of the apoptosis-inducing effect of pGenesil-siSurvivin. Thus, these results indicate that pGenesil-siSurvivin is an effective reagent to induce apoptosis of HBV-containing hepatoma cells.

### Targeted delivery of shRNA by jetPEI-Hepatocyte reagent

With the effectiveness of the generated shRNAs to inhibit HBV replication and induce apoptosis of hepatoma cells, we set out to identify reagents that could improve tissue targeting specificity; the latter is a prerequisite for clinical application of shRNA therapeutics. Specific targeting can reduce non-specific side effects of shRNA therapy and improve therapeutic effects by enriching shRNA production inside hepatocytes. We chose to target shRNA specifically to hepatocytes through tissue-specific plasma membrane receptor. For this, we tested jetPEI-Hepatocyte, a transfection reagent that binds to the unique hepatocyte receptor ASGPR.

**Figure 6:**
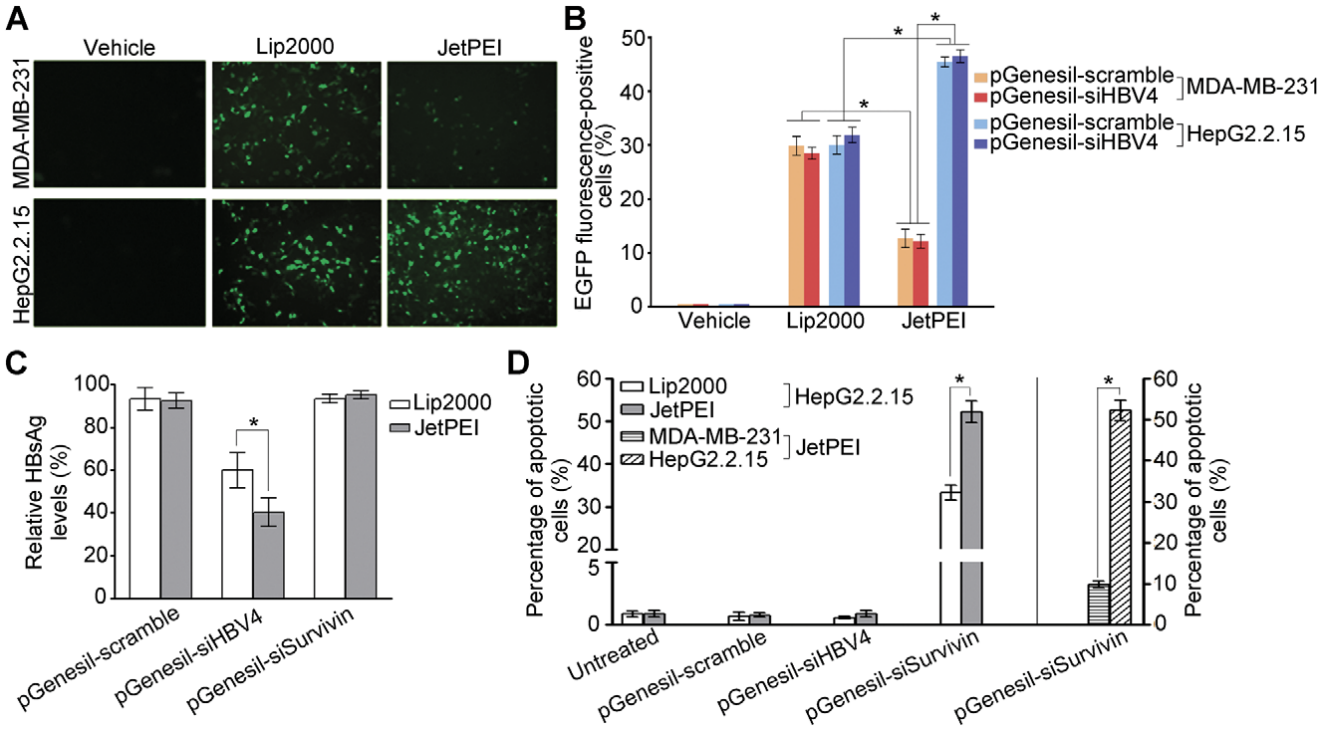
The jetPEI-Hepatocyte reagent mediates specific shRNA delivery into HepG2.2.15 cells. (**A**) Representative images for EGFP expression in the cells that are transfected with pGenesil-1. Vehicle control, Lipofectamine 2000 (Lip2000) and jetPEI-Hepatocyte (JetPEI) reagents were used to mediate transfection. HepG2.2.15 and MDA-MD-231 cells were imaged by fluorescent microscopy at 24-h post-transfection. (**B**) Percentages of EGFP-positive cells detected by flow cytometry at 24-h post-transfection. HepG2.2.15 and MDA-MD-231 cells were transfected with pGenesil-scramble and pGenesil-siHBV4 respectively. Three reagents were used for transfection: vehicle control, Lip2000, and JetPEI. (**C**) The levels of the HBsAg protein in culture medium normalized to the level of untreated cells at 72 h post-transfection. HepG2.2.15 cells were transfected with pGenesil-scramble, pGenesil-siHBV4, and pGenesil-siSurvivin respectively using either Lip2000 or JetPEI. (**D**) Percentages of apoptotic cells detected by flow cytometry at 48 h post-transfection. HepG2.2.15 cells were transfected as in (C) (the left side of the composite figure). MDA-MB-231 and HepG2.2.15 cells were transfected with pGenesil-siSurvivin by JetPEI (the right side of the figure). Data in (B), (C), and (D) are shown as mean ± SD of three independent experiments (*: P<0.01).

We transfected both HepG2.2.15 cells and a human breast cancer cell line, MDA-MB-231 cells, with pGenesil-scramble and pGenesil-siHBV4, respectively. While vehicle control transfection was not able to deliver the plasmids into these cells, Lipofectamine 2000 and jetPEI-Hepatocyte resulted in expression of the marker gene EGFP in both cell lines (Figure 6A and 6B). However, unlike Lipofectamine 2000, which leads to equivalent transfection efficiency in both cell lines (∼30%), jetPEI-Hepatocyte gives significant lower transfection efficiency in MDA-MB-231 cells (∼12%) but significant higher efficiency in HepG2.2.15 cells (∼45%) (Figure 6B). The more than 3 fold difference in transfection efficiency between HepG2.2.15 and MDA-MB-231 cells indicates that jetPEI-Hepatocyte is rather specific in targeting shRNAs into hepatocytes. We further tested the transfection efficacy of jetPEI-Hepatocyte for other types of cells. Consistently, jetPEI-Hepatocyte resulted in low efficiency in transfecting other non-hepatocyte cells, including the human prostate cancer cell line PC3, the lung adenocarcinoma epithelial cell line A549 and the cervical cancer cell line HeLa cells (Figure S2). On the contrary, jetPEI-Hepatocyte led to effective transfection in additional hepatoma cell lines Hu-7 and Hep3B (Figure S2), like in HepG2.2.15 cells. Thus, these data clearly demonstrate the relative specificity of jetPEI-Hepatocyte to hepatocytes.

We then tested whether the improved transfection specificity and efficiency brought by jetPEI-Hepatocyte could lead to increased inhibitory effects of the shRNAs. First, when pGenesil-siHBV4 was transfected into HepG2.2.15 cells, compared to Lipofectamine 2000, jetPEI-Hepatocyte resulted in a 1.5-fold more decrease of the secreted HBsAg protein level (Figure 6C). Second, jetPEI-Hepatocyte increased the rate of pGenesil-siSurvivin-induced tumor cell apoptosis to 52%, significantly higher than 33% caused by Lipofectamine 2000 (Figure 6D). The apoptosis effect induced by pGenesil-siSurvivin is specific as pGenesil-siHBV4 and pGenesil-scramble induced only ∼0.8% of apoptotic rate. Last, using jetPEI-Hepatocyte, pGenesil-siSurvivin only induced 9.8% of apoptotic rate in MDA-MB-231 cells, while causing 52% in HepG2.2.15 cells (Figure 6D). Thus, jetPEI-Hepatocyte leads to rather specific delivery of shRNAs into hepatocytes and enhances the protective effects of pGenesil-siHBV4 and pGenesil-siSurvivin.

### Synergistic effects of pGenesil-siHBV4 and pGenesil-siSurvivin in inducing apoptosis of hepatoma cells

Recent studies have deepened our understanding of the role of HBV in hepatoma development. Among these is the finding that the viral protein HBx binds survivin indirectly through HBXIP and increases the protein level of survivin, leading to an anti-apoptosis effect in hepatoma cells [16], [18], [19]. Indeed, it has been shown that anti-HBx siRNA can reduce the survivin protein level [18]. In our research, we show that the survivin mRNA level is downregulated by pGenesil-siSurvivin to 40.98% of its level in untreated cells. We hypothesized that if we combined downregulation of survivin at the mRNA level by pGenesil-siSurvivin and at the protein level by an anti-HBx shRNA, we might be able to achieve enhanced effects in reducing the survivin protein level and inducing apoptosis of hepatoma cells.

**Figure 7:**
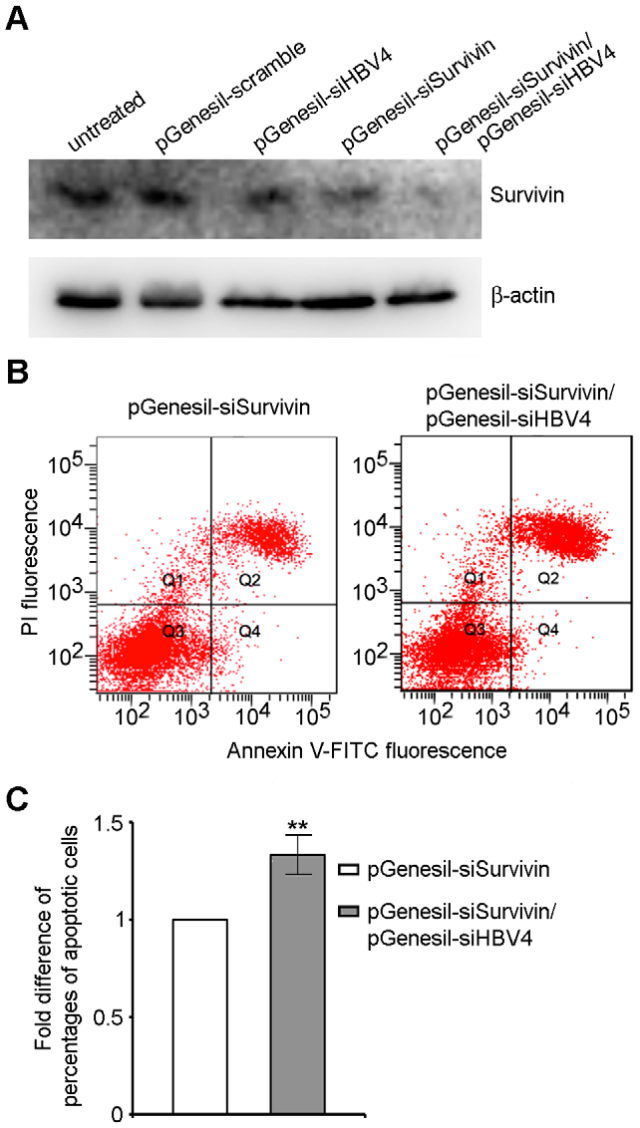
pGenesil-siSurvivin and pGenesil-shiHBV4 have synergistic effects in decreasing the survivin protein level and inducing apoptosis of HepG2.2.15 cells. HepG2.2.15 cells were transfected with pGenesil-scramble, pGenesil-siHBV4, pGenesil-siSurvivin and pGenesil-siSurvivin/pGenesil-siHBV4, respectively. (**A**) Western blotting to detect survivin at 48-h post-transfection, with β-actin as loading control. (**B**) Annexin V-FITC and PI positive cells detected by flow cytometry at day 2 post-transfection. (**C**) Quantification of apoptotic cells shown in (B). Percentage of apoptotic cells is normalized to that of the cells transfected with pGenesil-siSurvivin. Data are shown as mean ± SD of three independent experiments (**: P<0.001).

To test this hypothesis, we transfected HepG2.2.15 cells with pGenesil-scramble, pGenesil-siHBV4, pGenesil-siSurvivin and pGenesil-siSurvivin/pGenesil-siHBV4, respectively. While pGenesil-scramble did not affect the protein level of survivin, pGenesil-siSurvivin reduced it as expected (Figure 7A). Consistent with a previous study [18], pGenesil-siHBV4 appeared also to reduce the survivin protein level, but to a much less extent. Notably, co-transfection of pGenesil-siSurvivin and pGenesil-siHBV4 was able to downregulate the survivin protein level to a greater extent than did pGenesil-siSurvivin alone (Figure 7A). Thus, pGenesil-siHBV4 has a synergistic effect with pGenesil-siSurvivin in downregulating the survivin protein level.

We next compared the efficacy of pGenesil-siSurvivin and pGenesil-siSurvivin/pGenesil-siHBV4 in inducing apoptosis of HepG2.2.15 cells. As shown in Figure 7B and 7C, co-transfection of pGenesil-siSurvivin/pGenesil-siHBV4 resulted in significant higher apoptotic rates of HepG2.2.15 cells, compared to transfection with pGenesil-siSurvivin alone. Thus, the new strategy established here to downregulate survivin at both the mRNA level by the anti-survivin shRNA pGenesil-siSurvivin and the protein level by the anti-HBx shRNA pGenesil-siHBV4 leads to enhanced apoptotic effects on hepatoma cells.

## Discussion

Utilization of RNAi to suppress gene expression has opened a new perspective in gene therapy. RNAi inhibits gene expression by degrading target mRNA in a sequence-specific manner [24]. In the present study, from six shRNA candidates, we identified pGenesil-siHBV4 as an effective shRNA that inhibits HBV replication both *in vitro* and *in vivo*. In addition, we generated pGenesil-siSurvivin that induces apoptosis of HBV-positive hepatoma cells. Importantly, to increase the specificity of shRNA treatment, we identified jetPEI-Hepatocyte for targeting shRNAs into hepatocytes, not other types of cells. Crucially, we established a novel approach to enhance hepatoma cell apoptosis via the synergistic effects of pGenesil-siSurvivin and pGenesil-siHBV4.

Currently, there are various systems of RNAi, including chemically synthesized siRNA, viral vector-mediated RNAi, pRNA (packaging RNA)/siRNA and plasmid-based expression system [14], [25], [26], [27], [28]. It has been shown that the mRNA silencing effects with plasmid-based RNAi last longer and are more stable and economical [25]. We therefore chose the pGenesil-1 plasmid expression system for generating shRNA. In addition, the efficacy of RNAi is highly dependent on its targeting site within the gene [29]. In fact, it has been shown that only 1 out of 5 designed siRNAs/shRNAs induces efficient gene silencing, possibly due to the secondary structural constraints of target mRNA [30], [31]. Thus, we designed six shRNAs that target six different regions in the HBV genome. Using HepG2.2.15 cells as an *in vitro* HBV infection model system, we demonstrate that pGenesil-siHBV4 is most effective in inhibiting HBV replication.

pGenesil-siHBV4 is designed to target a region in the HBV X ORF. The X gene encodes HBx which has 154 amino acids in length. The X ORF is partly overlapped by other ORFs and is transcribed independently of the other viral transcripts [32]. The HBV transcripts are usually unspliced and have a common end that includes the conserved HBx sequence (Figure 1A). This may give pGenesil-shiHBV4 high efficacy in inhibiting HBV replication. Notably, HBx affects a variety of cellular processes, including gene transcription, cell cycle progression, DNA damage repair, cell proliferation, apoptosis and induced cytokine expression, making it a good target for counteracting deleterious effects of HBV [33], [34], [35], [36]. Moreover, previous research suggests that HBx expression may play a role in HBV-mediated hepatocarcinogenesis [37]. It is possible that pGenesil-siHBV4 might be useful to inhibit HBV-induced HCC transformation. However, this notion needs to be tested using a model system by which chronic HBV infection leads to HCC.

To validate the HBV inhibitory effects of pGenesil-siHBV4 observed in cultured cells, we investigated its *in vivo* efficacy. Currently, models for studying HBV infection include duck (DHBV), woodchuck (WHV), acute mouse HBV infection and human HBV-infected chimpanzees, as well as HBV transgenic mice. Among them, the acute mouse HBV infection model is useful and economical in testing *in vivo* efficacy of anti-HBV reagents. In the present study, we thought to test the *in vivo* ability of pGenesil-siHBV4 in inhibiting HBV replication and therefore chose to use the acute mouse HBV infection model by hydrodynamic mouse tail vein injection, which introduces a large amount of saline solution equivalent to 10% of mouse body mass within 5 to 8 seconds. It has been proposed that the large volume injected with high rate into blood circulation leads to blood retro-flow into the liver and the DNA solution is likely to accumulate in the inferior vena cava [38]. As a result, such hydrostatic pressure forces DNA plasmids to enter hepatocytes through membrane pore [39]. Consistently, our data indicate that tail vein hydrodynamic injection successfully delivers the HBV plasmid to the liver tissue, shown by the fact that EGFP signal is most evident in the liver among mouse internal organs. Moreover, the HBs and HBe antigens are readily detectable after injection of the HBV plasmid. Importantly, our data indicate that when injected, pGenesil-siHBV4 is able to inhibit HBV replication in the liver tissue, shown by significant decrease of both the mRNA and protein levels of HBsAg and HBeAg, decreased HBcAg expression in the liver hepatocytes, and reduced HBV cccDNA in the liver. These results suggest that pGenesil-siHBV4 is a promising shRNA therapeutics for HBV infection. It is worth to note that to test the efficacy of pGenesil-siHBV4 against chronic HBV infection, future studies using chronic HBV infection models, such as HBV transgenic mice, may be needed. That way, the dose and frequency of shRNA injection can be determined to ensure optimized inhibition of persistent HBV replication.

Because nearly 25% of chronic HBV infections terminate in hepatocellular carcinoma, we also identified another shRNA, pGenesil-siSurvivin, to induce apoptosis of HBV-positive hepatic tumor cells. Survivin is one of the most tumor-specific genes of the vast majority of cancers and is scarcely expressed in normal adult tissues [40]. Moreover, survivin functions in inhibiting tumor cell apoptosis. Accordingly, downregulation of survivin by siRNA has been shown to induce apoptosis of some tumor cells [14]. Here, we demonstrate that the survivin shRNA pGenesil-siSurvivin is able to induce HepG2.2.15 cell apoptosis.

To maximize the apoptosis of hepatoma cells, we combined two different mechanisms to downregulate survivin. It has been shown that the HBV protein HBx increases the protein level of survivin. Accordingly, downregulation of HBx can reduce the survivin protein level. Consistently, when HepG2.2.15 cells were co-transfected with pGenesil-siSurvin and pGenesil-siHBV4, we not only observed much less protein level of survivin compared to transfection with pGenesil-siSurvivin alone, but also achieved significantly higher apoptotic rates of the cells. One puzzle that is currently not understood is how HBx increases the survivin protein level, even though HBx is demonstrated to bind survivin through a bridge protein HBXIP. Some evidences suggest that such effect of HBx may be mediated by a protein called hepatoma upregulated protein (HURP) [19]. Thus, there may be other strategies to further optimize downregulation of survivin once we understand more in detail of the molecular events related to HBx and survivin. As we could not reach 100% hepatoma cell death in the present study, other possible approaches need to be explored in future studies. Nevertheless, our data established for the first time a new strategy to use two shRNAs with synergistic effects to target a key mechanism by which HBV facilitates hepatoma development through upregulation of survivin.

One important aspect of using shRNA as therapeutics is specific delivery of shRNA into the targeted cells, in order to minimize unwanted shRNA effects to other cells. To achieve this goal, some major approaches have been developed, including cationic polymer- or liposome-assembled cellular proteins, RNA aptamer, protamine-fused Fab fragments and the formation of different therapeutic nanoparticles containing siRNA [7], [14], [26], [41], [42], [43]. In these protocols, shRNAs are targeted to specific cell types via binding to cell plasma membrane receptors, followed by endocytosis. Here, we have identified jetPEI-Hepatocyte, a galactose-conjugated polyethylenimines, as a targeting reagent for specific delivery of shRNAs into hepatocytes. This strategy is based on the fact that ASGP receptors that the jetPEI-Hepatocyte binds to are specifically expressed in hepatocytes [44], [45]. Our data show that jetPEI-Hepatocyte is rather effective in delivering these shRNAs to heptoma cells, instead of other types of cells. Furthermore, jetPEI-Hepatocyte enhances significantly the effects of these shRNAs via increased delivery efficiency to HepG2.2.15 cells.

Taken together, our data indicate that pGenesil-siHBV4 effectively inhibits HBV replication and that pGenesil-siSurvivin results in apoptosis of HBV-positive hepatoma cells. Moreover, we improved their *in vitro* efficacy and tissue specificity via jetPEI-Hepatocyte mediated specific targeting to hepatocytes. These shRNAs delivered by jetPEI-Hepatocyte, especially the combination of the anti-HBx shRNA pGenesil-siHBV4 and the anti-survivin shRNA pGenesil-siSurvivin, are promising therapeutics for potential application in the two stages of the detrimental disease – chronic HBV infection and its related HCC.

## Materials and Methods

### shRNA plasmid construction

Six target sites of HBV shRNAs were selected from the conserved regions of the HBV genome (GenBank accession No. U95551) to target different gene regions and avoid homology to human mRNA sequences. The synthesized DNA oligonucleotides all contain 5′-BamHI-CC-(19-nucleotide sequence in sense direction)-(9-nucleotide loop: TTCAAGAGA)-(19-nucleotide sequence in antisense direction)-TTTTT-HindIII-3′ (synthesized by Invitrogen, Carlsbad, CA). The CC sequence facilitates efficient initiation of transcription and the TTTTT signal is required for transcription termination of RNA polymerase III (Genesil Corp., Wuhan, PR China). The DNA oligonucleotides were annealed and subcloned into the cohesive ends of the BamHI/HindIII-digested plasmid pGenesil-1. The plasmid pGenesil-scramble contains an irrelevant DNA sequence as control. The plasmid pBluescript-HBV contains the full length HBV genomic DNA (subtype ayw).

### Cell line and transfection

HepG2.2.15 cells (gift of Mengji Lu and Michael Roggendorf, Institute of Virology, University Hospital of Essen, Essen, Germany; [46]) and MDA-MB-231 cells (China Center for Type Culture Collection, Wuhan, PR China) were cultured and maintained in Dulbecco's Modified Eagle medium (DMEM, Invitrogen, Carlsbad, CA), supplemented with 10% heat-inactivated fetal bovine serum (FBS), 100 U/L penicillin and 100 U/L streptomycin. All cultures were maintained at 37°C in a moist atmosphere containing 5% CO_2_. Cells were seeded in 24-well culture plates at 24 hours before transfection. Cell transfection was conducted according to the manual of Lipofectamine 2000 (Invitrogen, Carlsbad, CA).

### Fluorescent microscopy and flow cytometry

At 24 hours post-transfection, fluorescent images were captured by a fluorescent microscope. Random fields were photographed with same exposure time. The *in vivo* EGFP signal was detected by KODAK image station 4000 MM (School of Basic Medical Science, HUST, Wuhan, PR China) and stereoscopic microscope (National Laboratory for Optoelectronics, HUST, Wuhan, PR China). The transfected cells and single cell suspension of the liver tissue were washed twice with ice-cold PBS, and then analyzed for EGFP signal by flow cytometry using FACScalibur (Becton Dickinson, Franklin Lakes, NJ). For detecting apoptosis, cells were further incubated with 10 µg/mL annexin V-FITC and 50 µg/mL propidium iodide at room temperature for 20 min before flow cytometry assay.

### RNA isolation and SYBR green real-time PCR assay

Total RNA was extracted with Trizol Reagent (Invitrogen, Carlsbad, CA) according to the manufacturer's protocol. The first strand cDNA synthesis kit - ReverTra Ace-α-TM (Toyobo, Osaka, Japan) was used for reverse transcription. Equal amounts of cDNA were subjected to PCR, in the presence of the SYBR green dye with the QuantiTect SYBR Green RT-PCR Kit (Qiagen, Beijing, PR China), and detected by the ABI PRISM 6700 Real-Time PCR System (Applied Biosystems, Foster City, CA). The primers for HBx are 5′-CTC TCT GCA ATG TCA ACG A-3′ (F) (GenBank accession number: U95551; position: 1673–1691) and 5′-TTA TGC CTA CAG CCT CCT A-3′ (R) (GenBank accession number: U95551; position: 1794–1776). The primers for HBc are 5′-GTC TTT CGG AGT GTG GAT TC-3′ (F) (GenBank accession number: U95551; position: 2262–2281) and 5′-ACC TGC CTC GTC GTC TAA C-3′ (R) (GenBank accession number: U95551; position: 2365–2341). The primers for HBs are 5′-TCA CAA TAC CGC AGA GTC-3′ (F) (GenBank accession number: U95551; position: 233–250) and 5′-ACA TCC AGC GAT AAC CAG-3′ (R) (GenBank accession number: U95551; position: 382–365). The primers for survivin are 5′-CAC CAC TTC CAG GGT TTA TTC C-3′(F) and 5′-TCT CCT TTC CTA AGA CAT TGC TAA GG-3′(R). The β-actin primers are 5′-CCT AGA AGC ATT TGC GGT GG-3′(F) and 5′-GAG CTA CGA GCT GCC TGA CG-3(R)′. PCR was performed with 40 cycles of 15 seconds at 95°C, 15 seconds at 60°C and 15 seconds at 72°C. The results are normalized to the internal control β-actin and the mRNA level is expressed as a relative ratio of Ct (the PCR cycle number at threshold): Ct of target gene mRNA/Ct of β-actin mRNA.

### ELISA and TRFIA assay of viral proteins

The viral proteins HBsAg and HBeAg from cell culture medium or mouse serum were measured using ELISA according to the manufacturer's protocol (KeHua Bio-tech, Shanghai, PR China). The absorbance was measured at 450/630 nm (A450/650) using an ELISA Reader (TECAN,Salzburg, Austria). In the dose-response experiment, mice were injected with 5, 10, 15, 20, 25, or 30 µg of pBluescript-HBV, and the sera were separated and assayed by time-resolved fluoroimmunoassay (TRFIA) for HBsAg and HBeAg at day 4 post-injection.

### Mouse model and hydrodynamic injection

For all *in vivo* experiments, we used 4- to 6-week-old BALB/C male mice with weight of 20–25 g (the Laboratory Animal Center of Huazhong University of Science and Technology, Wuhan, PR China). Animal experiments used in this study were approved by the Ethical Committee of Tongji Medical College. All experimental animals were housed in the specific-pathogen-free Laboratory Animal Center. Plasmids in PBS were injected into the tail vein within 5–8 seconds. The injected volume is 10% of the mouse body mass (e.g., 2 ml for a mouse of 20 g). Fifteen mice were randomly divided into 3 groups (5 mice in each group): 1) HBV group: the mice were injected with 15 µg pBluescript-HBV; 2) pGenesil-scramble group: the mice were co-injected with 15 µg pBluescript-HBV and 10 µg pGenesil-scramble (pBluescript-HBV/pGenesil-scramble); and 3) pGenesil-siHBV4 group: the mice were co-injeceted with 15 µg pBluescript-HBV and 10 µg pGenesil-siHBV4 (pBluescript-HBV/pGenesil-siHBV4).

### Immunohistochemical staining

Four days after injection, mice were killed and liver tissues were fixed in 4% neutral buffered polyoxymethylene. Paraffin embedded tissue sections were subjected to immunohistochemistry. We used the mouse monoclonal anti-HBcAg antibody as the primary antibody (Santa Cruz Biotechnology, Santa Cruz, CA). Immunostaining results were evaluated by calculating the mean density using the Image-Pro Plus software. (Mean density = IOD/Area; IOD: integrated optical density)

### JetPEI-Hepatocyte transfection

For transfection mediated by jetPEI-Hepatocyte, cells were seeded in 24-well culture plates at 24 hours before transfection. The jetPEI-Hepatocyte/DNA complexes were prepared by adding 1 µg of plasmids and 3.2 µl of jetPEI-Hepatocyte reagent into 50 µl of 150 mM NaCl. The mixed complexes were incubated for 15–30 minutes at room temperature and then added onto cells.

### Western blotting

Whole cell lysates were prepared from HepG2.2.15 cells at 48-h post-transfection and separated by SDS- PAGE at 100 V for 1 hr. Separated proteins were then transferred to PVDF membranes (Millipore, Billerica, MA). The membranes were blocked in 5% nonfat dry milk in PBS containing 0.1% Tween-20 for 2 hr at room temperature. Then the membranes were incubated with the anti-survivin antibody or the anti β-actin antibody (Santa Cruz, Santa Cruz, CA) overnight at 4°C. The membranes were washed three times and incubated with HRP-conjugated secondary antibodies. Proteins were visualized by ECL Western blotting substrate (Themo Pierce, Rockford, IL).

### Quantification of HBV DNA

The culture media were collected at various time points after transfection. The HBV DNA copies were determined by quantitative real-time PCR analysis using the Quantification Kit of Hepatitis B Virus DNA (KeHua Bio-tech, Shanghai, PR China). PCR was done according to the kit manual and the primers for PCR were 1) HBV forward: 5′-CAG GTC TCT GCC AAG T-3′ and 2) HBV reverse: 5′-TGC GGG ATA GGA CAA C-3′. A series of dilutions of known concentrations of HBV DNA were used as control to establish the linear standard curve.

### Quantification of HBV cccDNA

The nuclear HBV cccDNA was extracted according to the procedures described previously [47], [48]. Briefly, HBV-infected mouse liver cells were collected at 96 h after hydrodynamic injection, lysed in 500 µL of cell lysis buffer, and centrifuged for 10 min at 12,000 rpm at 4°C. Then, the precipitates were treated with 500 µL of nuclear lysis buffer and incubated for 30 min at 37°C. The lysates were neutralized with potassium acetate (pH 4.8) and then centrifuged for 20 min at 12,000 rpm at 4°C. HBV cccDNA was extracted from the supernatant with phenol/chloroform, precipitated with ethanol, and dissolved in 50 µL of Tris-HCl (10 mM and pH 7.5) and 1 mM ethylenediaminetetraacetic acid. HBV cccDNA samples extracted from liver cells were treated for 1 h at 37°C with Plasmid-safe DNase (Epicentre Biotechnologies, Madison, WI) to eliminate the open circular duplex HBV DNA and single strand HBV DNA. HBV cccDNA was quantified by real-time PCR as described previously [48], [49], [50]. The primers used in the real-time PCR have been proved to specifically detect HBV cccDNA, but not the HBV genomic DNA, thereby excluding the possibility of detecting the injected HBV plasmid pBluescript-HBV [50]. The primer sequences are HBV cccDNA forward primer 5′-ACT CTT GGA CTC BCA GCA ATG-3′ and HBV cccDNA reverse primer 5′-CTT TAT ACG GGT CAA TGT CCA-3′. The PCR products were amplified using the SYBR Green PCR Master Mix (Roche, Shanghai, PR China). The PCR cycling program consisted of an initial denaturing step at 95°C for 5 min, followed by 45 cycles of 95°C for 15 s and 61.5°C for 1 min.

### Statistical analysis

The data are presented in mean ± SD from three independent experiments. The data are analyzed by t-test for data of two groups and by ANOVA, Bonferroni post-hoc test, for data of more than two groups.

## Supporting Information

Figure S1 **EGFP-positive cells at 24-h post-transfection.** HEK 293 cells were transfected with pGenesil-1 using Lipofectamine 2000.
(TIF)

Figure S2 **EGFP-positive cells at 24-h post-transfection.** PC3, A549, HeLa, Hu-7, and Hep3B cells were transfected with pGenesil-1 using jetPEI-Hepatocyte.
(TIF)

Table S1 **The HBV mRNAs targeted by the anti-HBV shRNAs siHBV-1∼6 and the primers used for real-time PCR in** **Figure 2B** **.**
(TIF)
