# Supplementary figures and images for: Inhibition of Hepatitis B Virus and Induction of Hepatoma Cell Apoptosis by ASGPR-Directed Delivery of shRNAs
Source: PLoS One. 2012 Oct 19;7(10):e46096. doi: 10.1371/journal.pone.0046096 (PMC3477153; doi:10.1371/journal.pone.0046096)

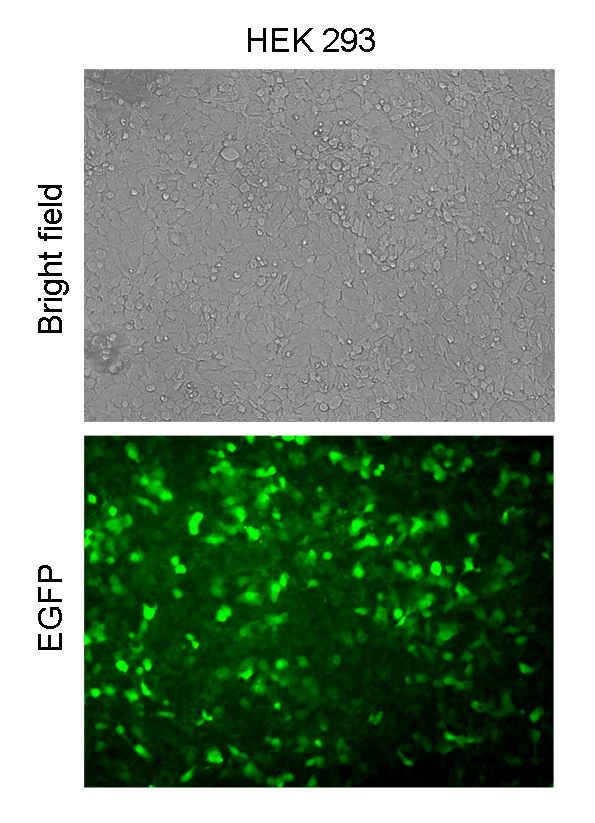

Supplement: Figure S1 — EGFP-positive cells at 24-h post-transfection. HEK 293 cells were transfected with pGenesil-1 using Lipofectamine 2000. (TIF) [file pone.0046096.s001.tif]

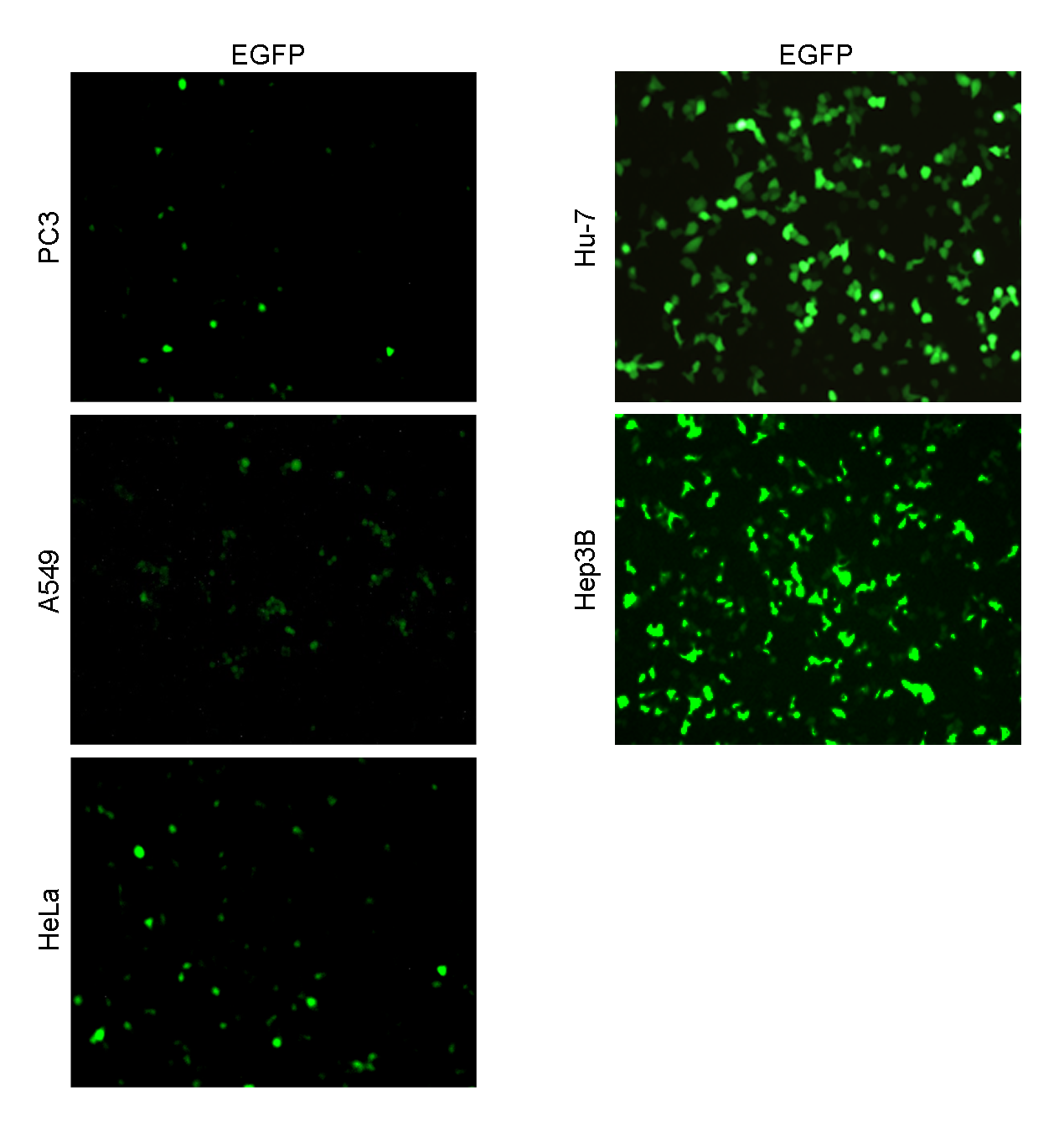

Supplement: Figure S2 — EGFP-positive cells at 24-h post-transfection. PC3, A549, HeLa, Hu-7, and Hep3B cells were transfected with pGenesil-1 using jetPEI-Hepatocyte. (TIF) [file pone.0046096.s002.tif]

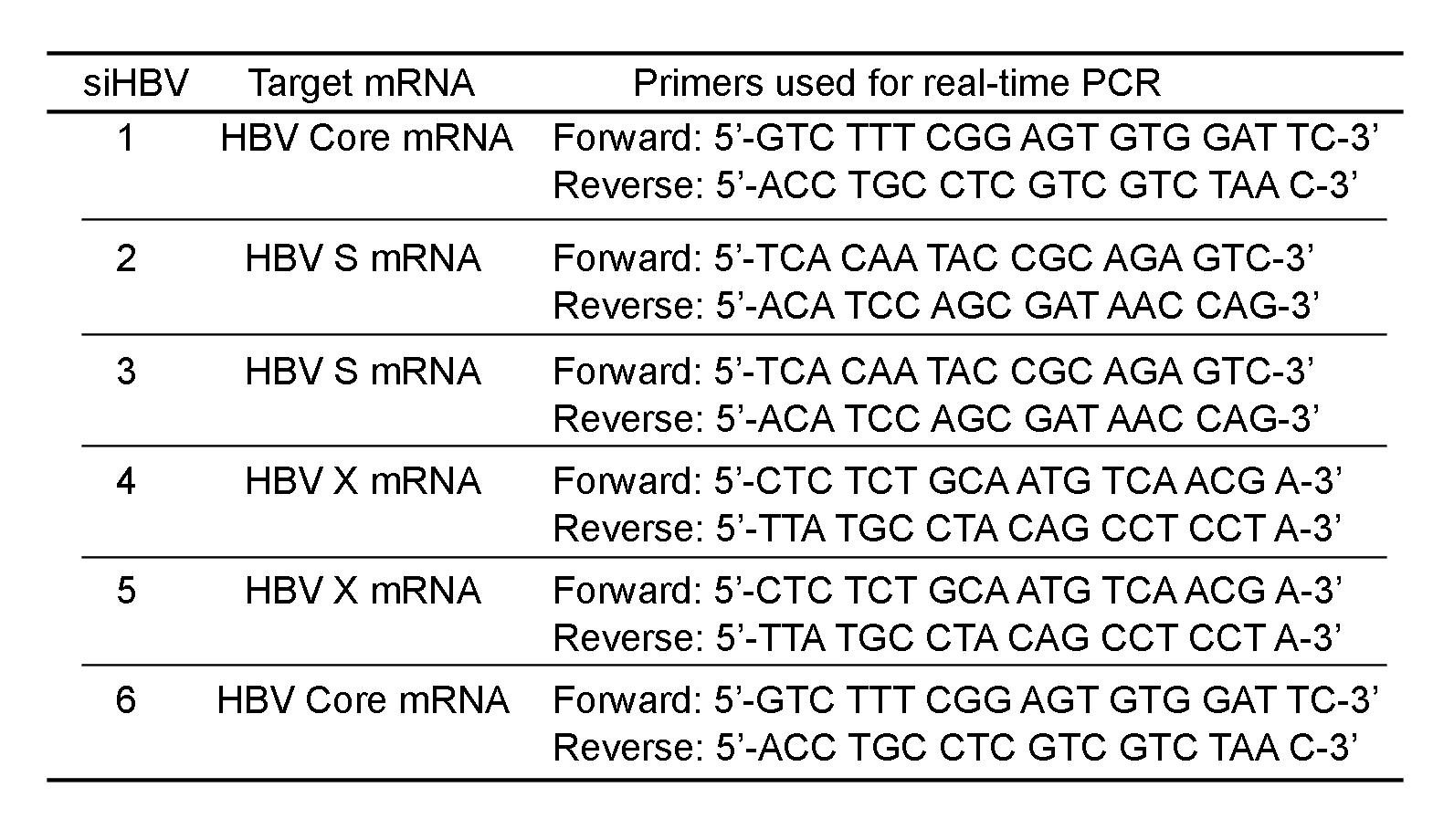

Supplement: Table S1 — The HBV mRNAs targeted by the anti-HBV shRNAs siHBV-1∼6 and the primers used for real-time PCR in Figure 2B . (TIF) [file pone.0046096.s003.tif]
